# Probing Mechanical Properties of Jurkat Cells under the Effect of ART Using Oscillating Optical Tweezers

**DOI:** 10.1371/journal.pone.0126548

**Published:** 2015-04-30

**Authors:** Samaneh Khakshour, Timothy V. Beischlag, Carolyn Sparrey, Edward J. Park

**Affiliations:** 1 School of Mechatronic Systems Engineering, Faculty of Applied Sciences, Simon Fraser University, Surrey, BC, Canada; 2 Faculty of Health Sciences, Simon Fraser University, Burnaby, BC, Canada; The University of Akron, UNITED STATES

## Abstract

Acute lymphoid leukemia is a common type of blood cancer and chemotherapy is the initial treatment of choice. Quantifying the effect of a chemotherapeutic drug at the cellular level plays an important role in the process of the treatment. In this study, an oscillating optical tweezer was employed to characterize the frequency-dependent mechanical properties of Jurkat cells exposed to the chemotherapeutic agent, artesunate (ART). A motion equation for a bead bound to a cell was applied to describe the mechanical characteristics of the cell cytoskeleton. By comparing between the modeling results and experimental results from the optical tweezer, the stiffness and viscosity of the Jurkat cells before and after the ART treatment were obtained. The results demonstrate a weak power-law dependency of cell stiffness with frequency. Furthermore, the stiffness and viscosity were increased after the treatment. Therefore, the cytoskeleton cell stiffness as the well as power-law coefficient can provide a useful insight into the chemo-mechanical relationship of drug treated cancer cells and may serve as another tool for evaluating therapeutic performance quantitatively.

## Introduction

Cytoskeletal proteins, inside the plasma membrane, are linked by molecular junctions which give the cell a complex and dynamic structure [1]. The cytoskeleton is responsible for cell growth, division, motility, and signaling, as well as the cell mechanical properties [2]. Since the cytoskeleton is the target of some anti-cancer drugs, these drugs can also influence its mechanical integrity [3], [4]. As anti-cancer drugs stiffen the cancer cells [5], quantifying mechanical properties of cancer cells exposed to chemotherapy can provide insight into the mechanistic action of drugs on cells which is important from two points of view. First, biochemical changes within the cell due to chemotherapy-induced cell death, such as actin reorganization, can be related to and quantified by the mechanical changes in cells [6]. Therefore, measuring mechanical changes such as the magnitude of cell stiffness allows for monitoring the drug effect [7]. Second, quantifying the deformability of cancer cells with respect to different dosages of chemotherapy can be helpful in further studying the vascular implications such as leukostasis that might arise from chemotherapy [5]. Therefore, mechanical characterization of cells may serve as an easier and faster quantitative indicator in evaluating therapeutic effects on cytoskeletal proteins, in comparison to biochemical fractionation and immunoblotting techniques.

The analysis of the drugs with less toxicity on normal cells is indispensable for curing the disease. Studying the effective concentration of drugs on different types of cancers has been extensively studied at the biochemical and molecular levels [4], [8], [9]. In order to combat cancer, an in-depth understanding of the dynamic functional processes such as cytoskeleton reorganization and mitotic changes are needed, which are available through both biochemical and mechanical cues. Therefore, integrating mechanical and physiological properties of cells can result in better understanding of the biophysical aspects of cancer. For example, the relationship between variations in cell stiffness and loading frequency has been used to quantify the health or integrity of a cell and is described by power-law rheology [10]. Many cell types have been characterized using a variety of stimulation methods in the literature. For instance, mouse fibroblast cells were measured with atomic force microscopy (AFM) [11], human bronchial epithelial cells were measured with magnetic twisting cytometry [12], kidney epithelial cells were measured with laser tracking microrheology [13], and mouse embryonal fibroblast cells were measured with a magnetic tweezer [14].

In this study, Jurkat cells, derived and immortalized from an acute lymphoid leukemia which is the most common type of blood cancer in children, was chosen as our demonstrative example [15]. Early treatment of the disease is essential, since the increased number of malignant cells could spread to other organs of the body. Previous studies have revealed the effect of artesunate (ART) on Jurkat cell apoptosis, while having modest side effects on normal cells [16]. There is an established overall relationship between cytoskeletal structure and cell mechanics as well; ART has been suggested to effect the cytoskeleton of Jurkat cells [17]. Thus, we hypothesize that quantifying the changes in the mechanical properties of Jurkat cells following exposure to ART utilizing optical tweezers and power-law rheology will provide the foundation for a new method of quantifying treatment efficacy. To accomplish this we defined a series of specific objectives as to 1) optimize an optical tweezer system to measure oscillation, 2) optimize a numerical model by reducing the number of free mechanical parameters, and 3) estimate key mechanical parameters by fitting the experimental data to the numerical model. The main contribution of this study is that, in our knowledge, it is the first work to apply the power-law theory to analyse alteration in mechanical properties of cancer cells exposed to a chemotherapeutic agent using oscillating optical tweezers. Specifically, by establishing the relationship between the Jurkat cell mechanics and ART dosages, the effect of the chemotherapy on the cells’ cytoskeleton stiffness and the power-law coefficient, which can be quantitative indicators of therapeutic efficacy, is demonstrated.

## Experimental Setup and Methods

### Experiment preparation

Jurkat cells (obtained from Dr. Robert D. Burke of University of Victoria) were cultured in RPMI-1640 supplemented with 1% penicillin and 10% FBS at 37°C in a humidified atmosphere of 5% CO_2_, and fresh culture medium were added every 2 to 3 days. After five passages, cells were cultured in four different dosages of ART (6.25, 12.5, 25, 50 μg/ml) for 24 hours. Microbeads were adhered to cell membranes and were used as a handle for cell manipulation [9]. Streptavidin-coated polystyrene beads (0.5 mg/ml) with radii of 1.55 μm (Bangs Lab, Fishers, IN) were washed in PBS three times and incubated with 0.4 mg/ml biotin-conjugated concanavalin A (Con A, Sigma) at 4°C for 40 minutes with gentle mixing. The Jurkat cells were washed in PBS three times and the antibody-coated beads were then rinsed and added to the washed Jurkat cell suspension.

For the purpose of imaging the variations in F-actin distribution via immunofluorescent microscopy, Jurkat cells were exposed to ART for 24 hours and were plated on Ploy-L-Lysine coated coverslips. First, the cells were washed in PBS. Next, they were fixed in 3.7% formaldehyde solution in PBS for 5 minutes and washed three times in PBS. They were then permeabilized with 0.1% Triton X-100 in PBS for 5 minutes and washed again in PBS. Finally, the cells were stained with 5 μg/ml fluorescent phalloidin conjugate solution in PBS (Sigma) for 40 minutes at room temperature and were washed in PBS several times to remove the unbound phalloidin conjugate. The images were obtained using a confocal microscope.

### Optical tweezer setup

**Fig 1:**
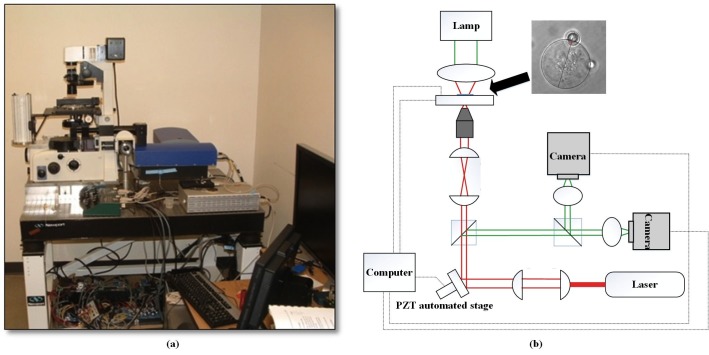
Oscillating optical tweezer: (a) experimental setup and (b) schematic of the optical tweezer.

The oscillating optical tweezer experimental setup (mmi Cell Manipulator, MMI AG, Zurich, Switzerland) is illustrated in Fig 1. A continuous wave 3W, Nd:YAG laser emitting light at a wavelength of 1064 nm was used with a Nikon TE2000 inverted microscope. A motorized beam expander was placed in the way of the laser beam in order to expand the beam size beyond the back aperture of microscope objective. Two galvanomirrors were driven by 2-D piezo-electric (PZT) stages, steering the laser beam. A dichroic mirror was used to reflect the laser beam into an oil immersion 100× objective and focus it on the sample. A 2-D motorized stage, driven by stepper motors with 78 nm positioning accuracy, was used to move the stage using visual feedback. A CCD camera and a CMOS camera were connected to the left side of the inverted microscope by utilizing a beam splitter. The CCD camera was used for monitoring the experimental process, while the CMOS high-speed camera (up to 500 fps) with a limited region of interest was used for bead motion tracking at high frequencies. All the optical and mechanical components were placed on an anti-vibration table (Newport Co.).

### System calibration

**Fig 2:**
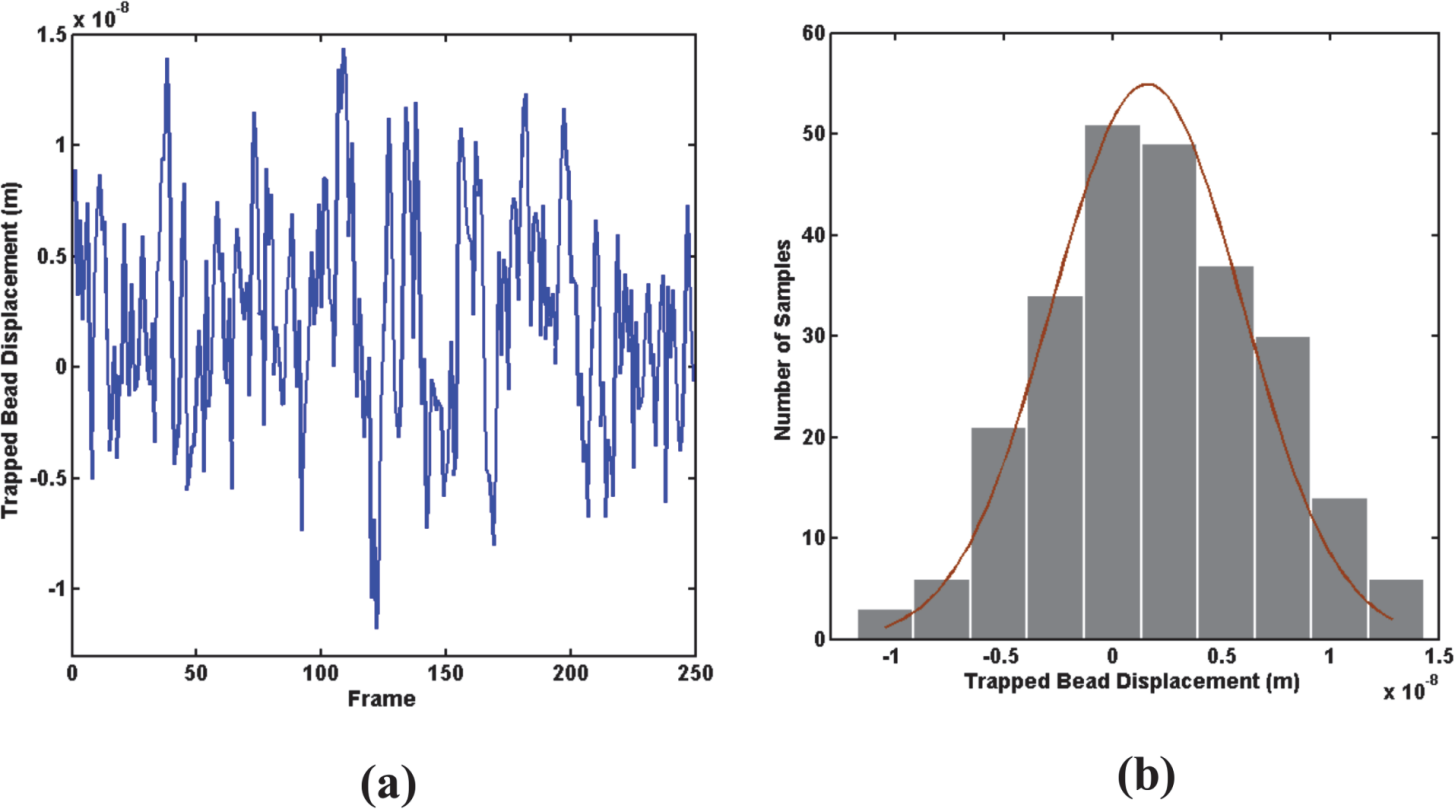
Measurement of trap stiffness: (a) Trapped bead displacement over 250 frames and (b) normal distribution of the bead displacement variation.

The calibration of the optical tweezer is an essential step in evaluating the force applied to the microbead. The first step in calibrating the system was determining the trap stiffness constant, *k*_*OT*_, of the optical tweezer. Utilizing the stiffness constant and bead displacement measurement, the optical tweezer force can be obtained. According to the equipartition theorem, the trap stiffness constant was calculated as follows [18], [19]:

$$\frac{1}{2}k_{OT}<x^{2}>=\frac{1}{2}k_{B}T$$ (1)

where *k*_*B*_ is the Boltzmann’s constant, *T* is the absolute temperature (300°*K*), *x* is the trapped bead displacement, and < > indicates a time average. The variance of optically trapped microbead positions determined the laser beam stiffness constant. After determining the trap stiffness (*k*_*OT*_), the trapping force of the optical tweezer was estimated as follows:

$$F=k_{OT}X$$ (2)

where *X* is the displacement of the trapped particle. Exposure time and frame rate of the CMOS camera were set to 1.25ms and 30 frames per second, respectively. The frame rate of the camera does not affect the stiffness constant measurement of the system. However, the exposure time affects the precision of the position measurement and, therefore, has an effect on the accuracy of the stiffness constant. The exposure time was determined after a series of experiments with the exposure time ranging between 0.1 ms and 30 ms. To determine the stiffness of the system, a microbead was trapped and the bead’s displacements was measured for 250 frames (Fig 2A). The variance of histogram of the trapped bead displacement (Fig 2B) was used to derive < *x* ^2^ >. The results were then used in Eq (1) to calculate the trap stiffness constant, *k*_*OT*_. The repeatability of this experiment was tested on 20 different beads.

### Oscillating cell-bonded microbeads using the optical tweezer

The cell manipulation process required anchoring a small region of the cell to the slide surface below. In order to attach the cells to the slide, the glass slides were coated with poly-L-lysine (Sigma). A microbead was trapped with the optical tweezer and attached to the surface of a cell. In order to test if the bead attachment was stable, the laser beam was shut off for several seconds. If the bead did not escape, it could be assumed that there was adherence between the cell and the bead. The beads that adhered to the Jurkat cell membranes were then used as a handle and trapped by the laser beam. Changing the angle of the galvanomirrors moved the trapped beads back and forth in a sinusoidal manner, which applied a time varying force on the cells. Note that the application of the time varying force was done by controlling the bead displacement in Eq (2). The oscillatory movement of the bead causes cytoskeletal deformation of the cell. The cell resists the deformation by producing internal stresses that are directly related to the cell mechanical properties [20]. Thus, cell mechanical properties could be calculated by applying a time varying force and measuring the resultant bead motion. The amplitude of the applied displacement oscillation remained constant during the whole experiment, and was equal to 0.437 μm. Jurkat cell mechanical responses were measured at different frequencies (0.1 Hz, 1 Hz, 10 Hz, and 100 Hz) of applied displacement. A total number of 79 cells were tested for the experiment. Four different dosages of ART treated cells (6.25, 12.5, 25, 50 μg/ml) and a control group of cells (each group employing between 15 and 17 cells) were chosen and the effect of sinusoidal forces on their mechanical responses were analyzed. To test the repeatability of the experiments, the bead displacement amplitudes were also measured for all groups over three different days.

## Data Analysis

**Fig 3:**
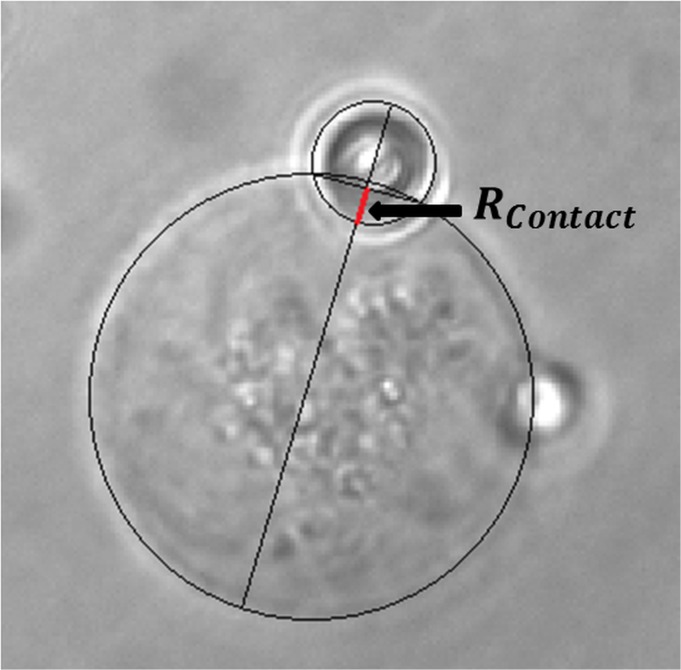
Sample cell-bead complex image with R-contact shown. The contact radius was determined by fitting a circle to the cell outline and another to the bead outline in the experimental images. A cord was drawn where the two circles overlapped. The distance from the bead circumference to the cord was defined as the contact radius.

For the analysis of the experimental data, the bead motion equation was used to calculate the cell mechanical properties in the frequency domain. The bead that was bound to the surface of the cell membrane was first trapped and then oscillated by the laser beam. The motion equation of the bead can be described by [21]:

$$mx^{\prime \prime }+6\pi \eta_{med}r_{bead}x^{\prime }+r_{cont}\eta_{cell}x^{\prime }+\frac{Y}{2r_{bead}^{2}}{\left(x-X_{0}\right)}^{\nu }+k_{OT}x=k_{OT}A\;\text{sin}\left(2\pi ft\right)$$ (3)

where *m* is the bead mass (*m* = 28.27 pg), *η*_*med*_ is the viscosity of the surrounding medium which is PBS (*η*_*med*_ = 10^−3^ *Pa*.*s*), *r*_*bead*_ = 1.55 μm is the polystyrene bead radius, and *r*_*cont*_ = 0.25~0.45 μm is the radius of the contact of the bead on the cell membrane, which is measured from the images of the experiment (Fig 3). The cells were assumed to have viscoelastic properties with a viscosity coefficient of *η*_*cell*_, while *Y* is a constant proportional to the stiffness of the cells. *X*_0_ is the displacement of the bead on the cell membrane caused by the fluid-like behaviour of the cell. *k*_*OT*_ is the spring constant of the optical tweezer which was calculated using the equipartition theorem, Eq (1). *A* and *f* are the amplitude (*A* = 0.437 μm) and frequency (*f* = 0.1, 1, 10, or 100 Hz) of the applied displacement oscillation, respectively. Finally, the exponent *ν* is related to the degree of non-linearity of the cell membrane’s material. In Eq (3), *Y*, *η*_*cell*_, *X*_0_, and *ν* are the unknown parameters that will be identified using the experimental results and an optimization approach presented below.

### Measurement of bead motion

In response to a time varying sinusoidal trapping force, the beads oscillated back and forth along with the oscillating force. The bead displacement was recorded by the CCD camera on the inverted microscope with a camera adapter of 1× magnification. The camera frame rate was set to 440 frames per second (fps).

The images were analysed using the Computer Vision System Toolbox in MATLAB to perform object detection, feature identification, and bead tracking. To do this, a system object for reading and displaying the video was created in the toolbox. Then, the first frame of the object was read and the region of the bead was selected, followed by applying the minimum eigenvalue corner detector of the toolbox to locate the corners of the bead. Finally, by using the Kanade-Lucas-Tomasi (KLT) point tracker, the point’s positions for all frames of the videos were tracked and used to measure the bead motion.

### Optimization

**Fig 4:**
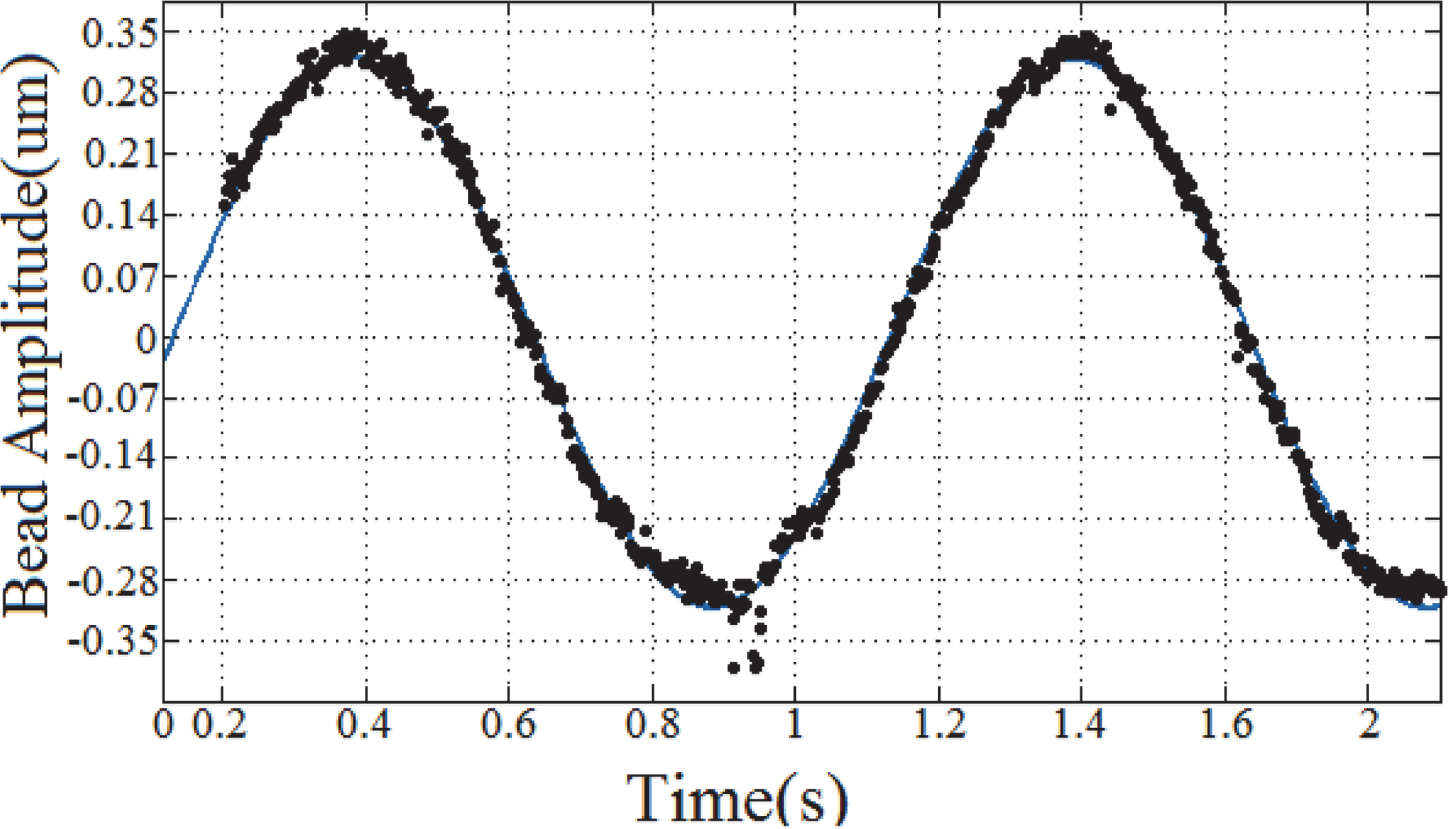
Sample curve fitting result for the bead’s oscillatory displacement for a 1 Hz applied force.

**Fig 5:**
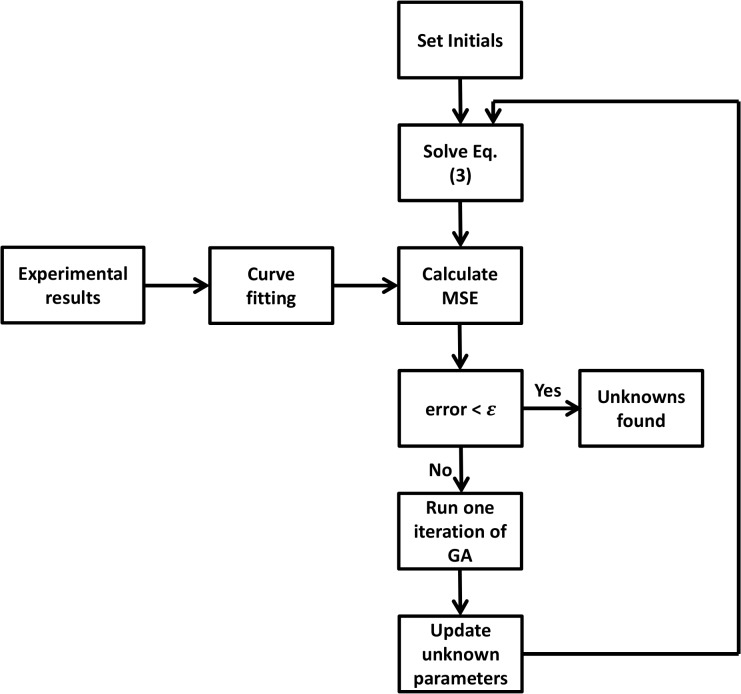
Cell model parameter identification algorithm (MSE: mean square error, GA: genetic algorithm).

The Curve Fitting Toolbox of MATLAB was used for fitting the experimental data. Using the least squares method, the best-fit curve of the bead displacement for each experiment was obtained. Fig 4 illustrates a sample result of the bead motion curve fitting. In order to identify the unknown parameters, *Y*, *η*_*cell*_, *X*_0_, and *ν* in Eq (3), the following procedure was carried out. First, Eq (3), which is a non-linear differential equation, was solved using the 4^th^ order Runge Kutta method. Then, a mean square error (MSE) approach was utilized as the regression tool for comparing the solution of Eq (3) and the best-fit curve of the bead displacement obtained from the experiment. Finally, an iterative genetic algorithm (GA) approach was used to minimize the error between the two curves by optimizing the unknown parameters. The flow chart of the cell parameters identification method is illustrated in Fig 5.

## Power-Law Rheology

According to Fabry et al. [9], various types of cells’ mechanical responses over a wide range of time scale are governed by a common behaviour that is called power-law rheology. The power-law rheology is based on the fact that, cell stiffness shows a power-law dependency on excitation frequency. The power-law rheology of living cells is related to the cell’s stiffness and power-law exponent. These two parameters are dependent on each other, where soft cells have a larger power-law exponent, which makes them to appear more fluid-like, while stiff cells have a smaller power-law exponent which makes them to appear more solid-like [10]. In our study, the power-law dependency of Jurkat cells exposed to ART was analyzed. Among the four different parameters of cells (*Y*, *η*_*cell*_, *X*_0_, and *ν*), *Y*, which is the coefficient proportional to the stiffness, changed according to a power-law, *f* ^*x*−1^, with respect to the changes in the applied force frequency.

### Finite element analysis

To test the accuracy of the derived material parameters in simulating the ART-treated Jurkat cell biomechanics, we modeled the displacement of the bead, which is fully bonded to the cell, under applied loads. The Jurkat cells were modelled as a sphere before optical manipulation using the COMSOL Multiphysics finite element analysis (FEA) software. The Coefficient Form PDE module, which has the following form, was applied for our equation based modeling:

$$e_{a}\frac{\partial^{2}u}{\partial t^{2}}+d_{a}\frac{\partial u}{\partial t}+\nabla .(-c\nabla u-\alpha u+\gamma )+\beta .\nabla u+au=f$$ (4)

All the parameters of Eq (4) were obtained based on the parameters of Eq (3), where c = *α* = *β* = *γ* = 0, *e*_*a*_ is equal to the bead mass, *d*_*a*_ and *a* are proportional to the viscosity and stiffness coefficients, respectively, and *f* is the applied force, and are defined based on the following equations:

$$d_{a}=6\pi \eta_{med}r_{bead}+r_{cont}\eta_{cell}$$ (5)

$$a=\frac{Y}{2r_{bead}^{2}}u^{2}$$ (6)

$$f=k_{OT}A\;\text{sin}\left(2\pi ft\right)$$ (7)

The bottom surface of the cell was fixed and the bead was fully bonded to the cell. The estimated material parameters for each Jurkat cell experiment for the different ART dosages were implemented for each simulation and the FE model geometry was adjusted to match the experimental contact radius for each simulation. Seventy nine models were constructed. Sinusoidal forces at different frequencies were applied on the bead corresponding to the experimental conditions, and the bead motion amplitude was determined from transient analysis of the model in COMSOL.

### Statistics

Analysing the statistics of the results has two objectives. The first one is to estimate the cell mechanical parameters *Y*, *η*_*cell*_, *X*_0_, and *ν*, whereas the second one is to find if the number of free parameters can be reduced. Four different models, which were obtained based on Eq (3), were analyzed. In the first model, all parameters were set free and there were no constraints. In the second model, *X*_0_ was held constant across the different ART dosages and the control group, while the other parameters remained as treatment-dependent. In the third model, *X*_0_ and *ν* remained constant for all cell conditions, while *η*_*cell*_ was set as treatment-dependent. In the last model, three parameters (*η*_*cell*_, *X*_0_, and *ν*) were constant across all cell conditions. As the changes in *Y* are considerably more significant than those of the others with respect to different cell conditions, *Y* was not held constant in any of these models.

## Experimental Results and Discussion

**Fig 6:**
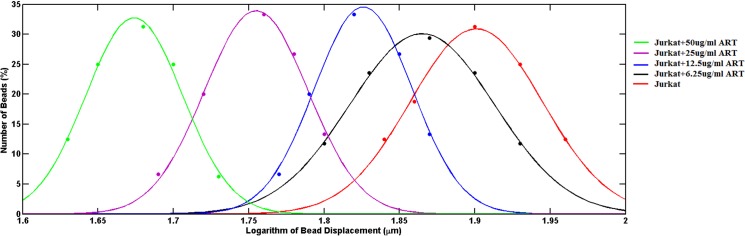
Probability function of the bead displacement amplitude for Jurkat cells treated with different dosages of ART, compared to the control group (dotted data: experimental results, solid lines: fitted curves).

The experimental results showed that, the bead displacement amplitude, which is inversely proportional to the cell stiffness, varied widely between the Jurkat cells of each group: (i) control–not treated with ART, (ii) treated with 6.25 μg/ml of ART, (iii) treated with 12.5 μg/ml of ART, (iv) treated with 25 μg/ml of ART, (v) treated with 50 μg/ml of ART. A histogram of all bead displacements revealed a log-normal distribution. The probability functions of the bead displacement amplitude for all cell conditions at 1 Hz frequency are illustrated in Fig 6. The median of bead displacement amplitude for the control group was 0.79 μm, with the standard deviation of 0.017 μm. Increasing the dosage of ART caused a decrease in the bead displacement amplitude. In the case of the groups of Jurkat cells treated with 6.25, 12.5, 25, and 50 μg/ml of ART, the medians of the bead displacement amplitude were 0.74 ± 0.016 0.66 ± 0.011, 0.57 ± 0.010, and 0.47 ± 0.008μm, respectively. The median values of the bead displacement amplitude decreased with the increase in the dosage as a result of the increase in cell stiffness. The results related to the repeatability of the experiment showed negligible changes in the standard deviation values, with the median value for each group not varying day to day.

The median of each cell group bead displacement along with the solution of Eq (3) were used based on the algorithm outlined in Fig 5 to identify a set of free parameters for each cell group. The goodness of the fit was satisfactory (*r* ^2^ = 0.95); however, we noticed that the degree of changes in three parameters, i.e. *η*_*cell*_, *X*_0_, and *ν*, were not comparable to the variation of *Y* at different cell conditions, for which *v* remained almost constant, *X*_0_ increased two-fold, *η*_*cell*_, increased three-fold; while *Y* increased by more than twenty-fold in comparing the drug treated cells to the control condition. Thus, these results indicate that the number of parameters could be reduced without decreasing the goodness of the fit.

## Model Parameter Fit

In order to identify the unknown parameters and find if the number of parameters can be reduced, four different statistical models were proposed in the Statistic section. The brief overview of these models are presented in Table 1. All four models, shown in Table 1, were compared using the F-test and the results are shown in Table 2.

**Table 1:** Four different statistical models to analyze cell parameters changes by changing ART dosages comparing to control group.

	*X*_0_	*ν*	*η*_*cell*_
**Model 1**	Free	Free	Free
**Model 2**	Constant	Free	Free
**Model 3**	Constant	Constant	Free
**Model 4**	Constant	Constant	Constant

*X*_0_: the displacement of the bead on the cell membrane caused by the fluidity of the cell, *v*: the degree of non-linearity of the cell’s material, *η*_*cell*_: cell’s viscosity coefficient

**Table 2:** Statistical results of four different models.

	Model 1	Model 2	Model 3	Model 4
*r* ^2^	0.95	0.95	0.96	0.95
*r*_*ss*_	0.391	0.888	0.427	0.819

*r*_*ss*_: Sum of squared residual

To test the independency of three parameters of *η*_*cell*_, *X*_0_, and *ν* to the drug treatment, the residual variance of fit of data to Eq (3) was analyzed. Thus, we have analyzed how well Eq (3) will be fitted to the median data, for each one of the four different models. The residual variance of fit of data for Model 1 and Model 3 were not significantly different from each other; therefore, Model 3 is preferred as the number of parameters is less than Model 1. The residual variance of fit of Models 2 and 4 were slightly larger than that of Models 1 and 3 (Table 2). Since Model 3 showed the best fit of the four different models, it can be concluded that the viscosity coefficient of the cells varied in response to the drug treatment and dosage. Finally, the estimated cell parameters, obtained by fitting Eq (3) to the experimental data under control conditions, as well as the Jurkat cells treated with the different dosages of ART, are shown in Table 3.

**Table 3:** Estimated cell parameters under the control condition and four different dosages of ART.

Cell Condition	*X*_0_ (nm)	*ν*	*η*_*cell*_ (Pa·s)
**Jurkat cells**	7.71 (sd = 1.03)	3.01 (sd = 0.013)	0.018 (sd = 0.013)
**Jurkat + 6.25 μg/ml ART**	6.85 (sd = 0.95)	3.02 (sd = 0.014)	0.044 (sd = 0.008)
**Jurkat + 12.5 μg/ml ART**	6.14 (sd = 1.2)	3.02 (sd = 0.018)	0.064 (sd = 0.009)
**Jurkat + 25 μg/ml ART**	5.29 (sd = 0.91)	3.05 (sd = 0.013)	0.069 (sd = 0.011)
**Jurkat + 50 μg/ml ART**	3.68 (sd = 1.3)	3.05 (sd = 0.019)	0.085 (sd = 0.015)

*X*_0_: the displacement of the bead on the cell membrane caused by the fluidity of the cell, *v*: the degree of non-linearity of the cell’s material, *η*_*cell*_: cell’s viscosity coefficient

**Fig 7:**
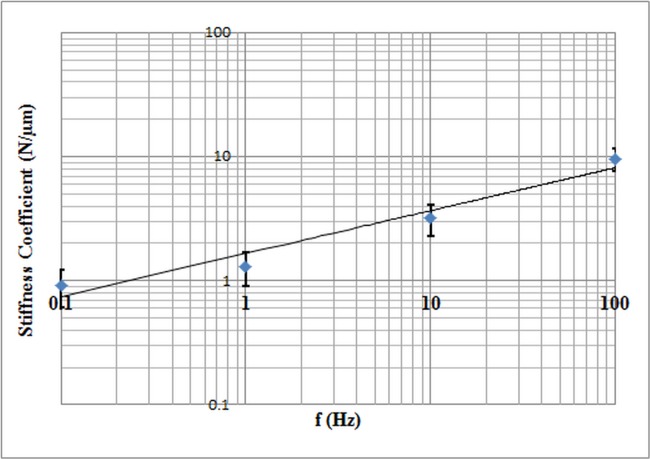
Stiffness versus frequency under the non-treated condition for frequencies between 0.1~100 Hz. Each data point represents the median value of 20 cells. *Y* increases with increasing frequency. Since the axes are logarithmic, the power-law dependency appears as a straight line with a slope of *x*—1. The power-law exponent is estimated to be 0.34, which shows a weak power-law dependency of *Y* on frequency.

**Fig 8:**
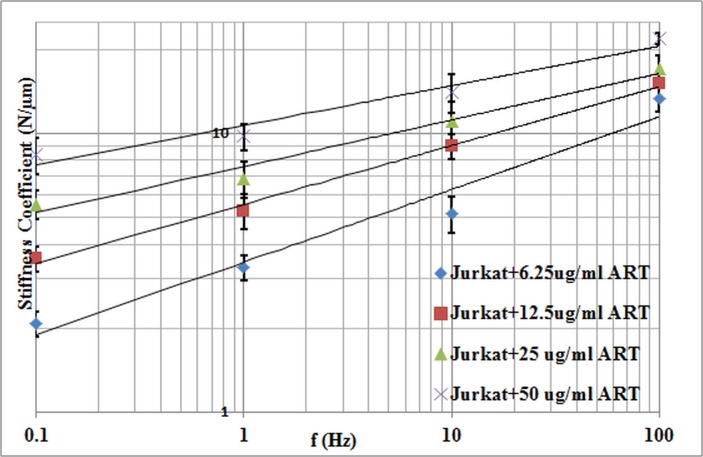
Stiffness coefficient versus frequency for Jurkat cells treated with different dosages of ART.

**Fig 9:**
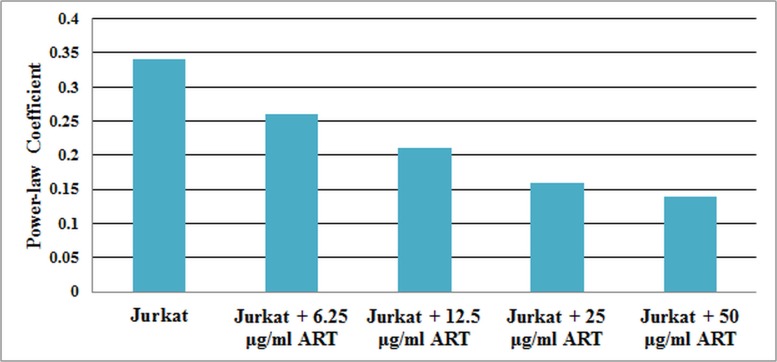
Power-law coefficient for non-treated cells and four different dosages of ART treated Jurkat cells.

Under control conditions there was a weak power-law dependency of *Y* on frequency (Fig 7). When the Jurkat cells were treated with ART, *Y* increased, while still maintaining a low power-law dependency on frequency (Fig 8). Increasing the dosage caused *Y* to further increase. The power-law dependency of the Jurkat cells treated with ART decreased with increasing dosage (Fig 9).

According to the results, the changes in cell stiffness and viscosity are dosage dependant, as shown in Table 2 and Figs 7 and 8. Both the stiffness and viscosity increased with increasing dosage. In order to test if there is a significant difference between different treatment dosages groups of cells, a MANOVA test was applied on different cell groups based on the stiffness and viscosity coefficients. Wilk’s Lambda measure was used to determine the F-value. Since, the estimated p-value was < 0.05, we can conclude that there is a significant statistical difference between the five different groups of cells. Moreover, the power-law coefficient, which is an intrinsic property of the cytoskeleton, decreased with increasing the dosage, which implies the cell structure transitioning from a liquid-like state to a solid-like state, according to Fabry et al. [10]. The results of the power dependence of stiffness are also in good agreement with previously reported studies, which used other measurement techniques [13], [10], [22], [23], [24].

Cai et al. [17] has reported an inhibition rate of more than 50% when the dosage of ART applied on Jurkat cells is more than 12.5 μg/ml, after 24 h exposure, and further increasing the ART concentration also decreased the cell growth rate significantly. Their results showed that ART can increase cell stiffness as well. Similarly, we observed a reduction in cell viability with increasing drug dosage, where the mechanism of cell death due to ART may be through apoptosis, which includes morphological changes such as cell shrinkage, blebbing, and stiffening. Cells with morphological changes were seen at all drug dosages, while the number of cells with these changes were increased with increasing drug dosage significantly.

The concentrations of ART that are selected in this study resulted in inhibition rate of > 50% [17]. A statistical analysis (ANOVA) was performed for studying the effect of different frequencies on cell stiffness at each group of cells. The power-law coefficient was decreased from 0.34 in the non-treated Jurkat cells to 0.14 in the cells treated with 50 μg/ml ART. The Jurkat cell’s mechanical properties such as stiffness coefficient and the power-law coefficient were changed significantly after the exposure to different dosages of the chemotherapeutic agent, which proves structural changes within the cells due to chemotherapy. These variations in stiffness and power-law coefficient at different drug concentrations may be related to the cytoskeleton reorganization.

**Fig 10:**
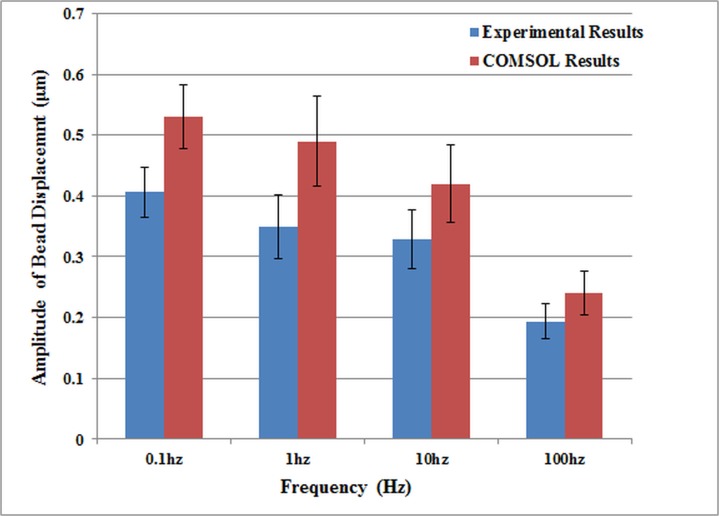
Comparison of the bead displacement amplitude between FEA and experimental results for non-treated cells.

**Fig 11:**
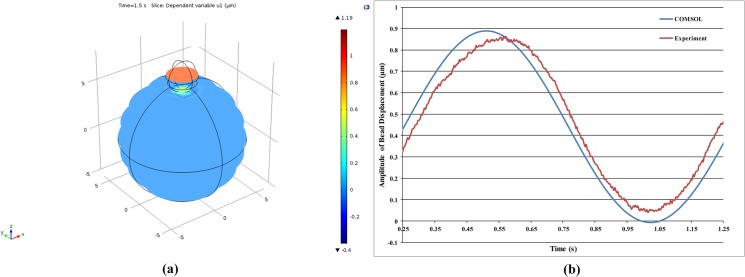
Sample results of FEA for the bead displacement amplitude over space and time of non-treated cells: (a) sliced plot of bead displacement amplitude over space, and (b) bead displacement amplitude over time (FEA versus the experimental results).

As mentioned previously, a FEA was carried out in COMSOL to test the validity of the derived material parameters in accurately simulate the ART-treated Jurkat cell biomechanics. As shown in Fig 10, the maximum amplitude of bead motion predicted by the FEA are in a good agreement with the experimental results, which verifies the accuracy of the proposed numerical cell parameters estimation method. The bead displacement over time predicted by the FEA also agreed with the experimental results (Fig 11).

**Fig 12:**
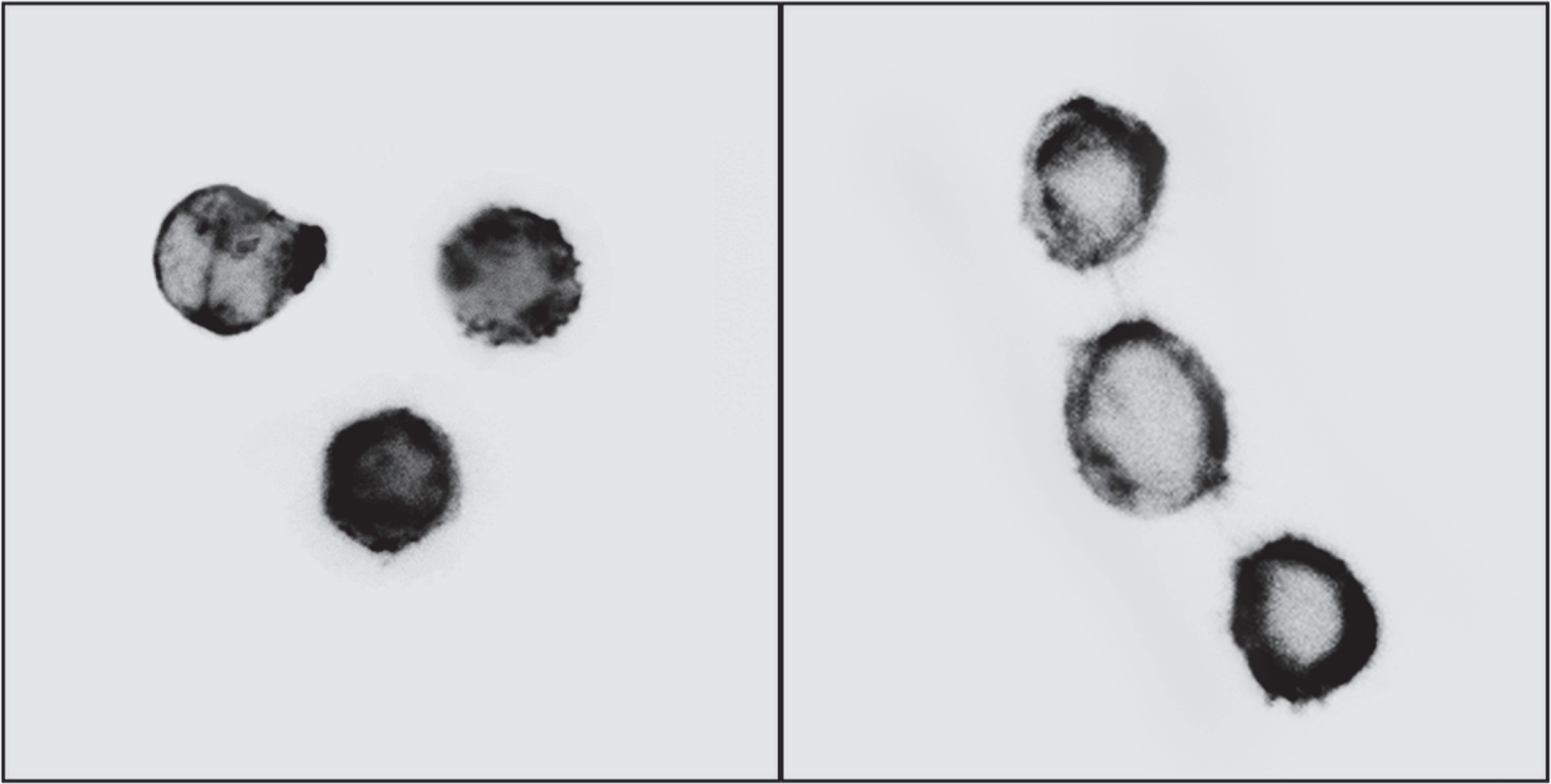
Sample immunofluorescnet images of the Jurkat cells (left: cells exposed to 50 μg/ml ART, right: non-treated cells).

The immunofluorescent microscope images of the non-treated cells and drug treated cells are shown in Fig 12. The results show a variation between F-actin structures of non-treated cells and drug treated cells. The F-actin distribution is larger in cells exposed to ART, compared to the non-treated cells, which is in agreement with the alterations in the mechanical properties between these two different cell groups. These results confirm the contribution of the F-actin in regulating the mechanical properties of drug treated cells.

The proposed method may serve as an easier and faster quantitative indicator in evaluating therapeutic effect on cytoskeletal proteins in comparison to biochemical fractionation and immunoblotting. However, there is a need to perform the analysis on variety of cell types and different chemotherapeutic agents before a generalized conclusion can be made. Also, the effect of chemotherapy may cause inter cellular mechanisms that may not directly affect the cell stiffness. For example, Li et al. [25] suggested that ART causes translocation of *β*-catenin and inhibition of unrestricted activation of Wnt/*β*-catenin pathway. Nevertheless, changes in cell mechanical responses due to drug effects are an indicator of cell behaviour deviation. Quantifying the behaviour deviation by performing mechanical characterization can be used as a complementary method to understand such responses. Therefore, the proposed method can potentially serve as a tool for a comprehensive cell mechanical characterization study with respect to various treatments.

## Conclusion

In this paper, the effect of different doses of ART on Jurkat cells stiffness and viscosity over four decades of frequency was analyzed. The stiffness of both untreated and ART-treated Jurkat cells increased with increasing frequency according to a weak power-law dependency. Moreover, the power-law coefficient decreased with increasing dose.

The results demonstrate that significant stiffening occurs in cancer cells after chemotherapy, which might be due to actin microfilament dynamic reorganization during apoptosis [26], [27]. This suggests that clinical observations may be performed with an oscillating optical tweezer combined with numerical simulations to quantify the mechanical properties of cancer cells following exposure to chemotherapeutic agents, in order to quickly assess the efficacy of the treatment. In addition, white blood cell stiffening after chemotherapy is a treatment complication that could lead to leukostasis and other vascular complications in some leukemia patients. Tracking the mechanical characteristics of white blood cells using optical tweezers may provide an early indicator of such complications. As a previous study [6] showed, the cytoskeleton reorganizes during the process of apoptosis induced by chemotherapeutic agents acting on leukemia cells. Therefore, measuring mechanical changes, such as the magnitude of cell stiffness, allows for monitoring the drug effect on cytoskeletal proteins as well. As a result, a powerful method for quantifying the effect of anti-cancer drugs and prevention of clinical complications following chemotherapy may be established by connecting cell mechanics with biological functions.
